# Book review of *An Introduction to MRI for Medical Physicists and Engineers*

**DOI:** 10.1002/acm2.13435

**Published:** 2022-01-19

**Authors:** Yanle Hu

**Affiliations:** ^1^ Ph.D., DABR, is an associate professor of Radiation Oncology at Mayo Clinic Arizona

## 1 OVERVIEW

Magnetic resonance imaging (MRI) is a noninvasive, nonionizing imaging modality that offers remarkable anatomic details and functional information of human beings and animals. It has been widely used in various fields such as healthcare to assist diagnosis and management of diseases and neuroscience to study how human brain functions. MRI theory and technology involve substantial amount of physics and signal processing. It is a challenging topic for medical physicists, engineers, and physicians. Even though various MRI textbooks already exist on the market, they are either technically oriented that are designed for scientists advancing MRI technology or clinically oriented that are suitable for physicians such as radiologists. There are few MRI textbooks specifically tailored to fit the needs of medical physicists and engineers. In 2019, Medical Physics Publishing published a new book, *An Introduction to MRI for Medical Physicists and Engineers*, to fill this gap.

The book was written by two MRI experts, Dr. Anthony B. Wolbarst and Dr. Nathan Yanasak. Dr. Wolbarst is a retired physicist who previously held teaching and research positions at Harvard Medical School and the National Cancer Institute, and served as a scientific manager at U.S. Environmental Protection Agency. Dr. Yanasak is an assistant professor in the department of Radiology and Imaging at Augusta University. The book offers a solid foundation for medical physicists and engineers who are interested in learning the basics of MRI theory as well as its hardware and quality assurance procedures.

## 2 PURPOSE AND AUDIENCE

The purpose of the book is to provide an introductory textbook of MRI science and technology. It is written at the beginning graduate level for medical physicists and engineers in training. The book does require reasonable knowledge of college physics and calculus. The more advanced topics, for example, Fourier transform and digital representation of signals and images, are introduced in the book as needed. The target audience of the book is medical physicists and engineers working in clinical settings as well as the graduate and postgraduate‐level trainees in these two fields. Other professionals, for example, scientists and physicians with an interest in MRI technology, may also find this book useful and informative.

## 3 CONTENTS AND HIGHLIGHTS

This book has 16 chapters and 299 pages. In my opinion, the book can be roughly divided into two parts: part one (Chapters 1–10) focused on establishing the theoretical basis to understand MRI and the other part (Chapters 11–16) focused on implementation and applications of MRI. The book starts with a brief introduction (Chapter 1) to nuclear magnetic resonance (NMR) phenomena, MRI, as well as a history of MRI. Next, it provides a quasi‐quantum description of NMR in a single voxel (Chapter 2). Even though quantum mechanics provides complete description of NMR, it can be overwhelming for medical physicists and engineers to master, especially at the beginning stage of their careers. Using a quasi‐quantum description allows good understanding of NMR concepts without getting into the daunting details of quantum mechanics. Chapter 3 introduces the concept of spatial encoding in a simplified one‐dimensional (1D) case, that is, how to differentiate magnetizations at different locations. It is spatial encoding that enables the evolution from NMR to MRI. In Chapter 3, it also introduces basic concepts and metrics that are commonly used to evaluate image quality, for example, resolution, contrast, noise, and signal‐to‐noise ratio. Chapter 4 focuses on magnetization of a voxel and introduces magnetization, thermal equilibrium, spin population dynamics, and relaxation. For a better understanding of MRI, it requires some basic knowledge of signal processing, which is the focus of Chapter 5. A classical approach to proton MRI is provided in Chapters 6 and 7, focusing on a single voxel and on a 1D case, respectively. To simplify the explanation, relaxation is temporarily left out. Chapter 7 introduces image reconstruction. But more importantly, it introduces the concepts of image space and k‐space, as well as the connection between the two. Chapter 8 provides an overview of MR hardware, including main magnet, gradient coils, transmit RF coils, and receiving RF coils. Chapters 9 and 10 are dedicated to relaxation mechanisms, that is, T1 and T2 relaxations, which are fundamental to the generation of various soft tissue contrasts. Starting from Chapter 11, the book shifts gears to implementation and applications. In Chapter 11, it introduces the spin‐echo pulse sequence, a basic MRI sequence that is commonly used to create MRI images. It discusses the selection of imaging parameters to generate different contrasts. In Chapter 11, it also covers the inversion recovery sequence, a variant of the spin‐echo sequence, which is designed to enhance T1 contrast or suppress signal from certain tissues such as fluid or fat. Chapter 12 covers creation of two‐dimensional (2D) MRI images, from excitation and selection of a 2D slice to signal spatial encoding, and to image reconstruction from the acquired signal. The explanation is based on a simplified 2D 4‐voxel (2 × 2) case, followed by a realistic example. The standard spin‐echo sequence is a good starting point for learning, but its acquisition speed can be very slow. Many techniques have been developed to expedite image acquisition. Chapter 13 covers various fast imaging methods including multislice imaging, fast spin echo, echo planning imaging, k‐space undersampling, non‐cartesian readout, and so on. These methods significantly improve the throughput of MRI and make it accessible to more patients. Chapter 14 touches more advanced topics, including magnetic resonance angiography, perfusion imaging, diffusion imaging, and functional MRI. These technologies greatly expand the capability of MRI and allow us to explore functionalities of the human body. Given MRI poses minimal risks (eligibility needs to be determined through appropriate MRI safety screening), it can be performed not only on patients but also on healthy human subjects because of its noninvasive and nonionizing nature. For medical physicists and engineers working on the MRI equipment, one essential responsibility is to ensure safe and efficient operation of MRI. The high magnetic field of MRI can be dangerous. MRI is safe only when all safety requirements are followed diligently. In Chapter 15, the book covers MRI safety information. It also discusses the required quality assurance procedures recommended by professional organizations like the American Association of Physicists in Medicine and the American College of Radiology. The book concludes with an overview of the exciting MRI technologies currently under active development, for example, quantitative imaging including MR fingerprinting, compressed sensing, and high field MRI. These are considered hot topics in MRI and may stimulate further interests from the audience.

## 4 CRITICAL ASSESSMENT

MRI being a powerful tool has already been widely used in diagnostic radiology and neurosurgery. Its usage in radiation oncology also keeps increasing. It is highly likely that medical physics and engineering professionals will encounter MRI at some point of their careers and want or need to have a better understanding of this fascinating technology. As the title states, *An Introduction to MRI for Medical Physicists and Engineers* is an introductory textbook for these professionals to learn basic MRI theory, implementation, and applications.

Although there are a few MRI textbooks in the market, they may not necessarily fit the needs of medical physicists and engineers. Clinically oriented textbooks may not have enough technical details or depths. General medical imaging textbooks designed for diagnostic physicists need to cover a variety of imaging modalities including X‐ray, fluoroscopy, CT, MRI, ultrasound, and so on. Therefore, they may not allocate enough space for the MRI technology. For example, the classical imaging physics textbook *The Essential Physics of Medical Imaging* by Bushberg et al. allocates only two chapters for MRI. On the other hand, textbooks designed for MRI scientists interested in advancing the MRI technology require substantial knowledge of quantum mechanics, which many clinical physicists and engineers may find intimating, especially many years after graduation. Considering medical physicists are heavily involved in patient healthcare and clinical investigations, they play a very important role in clinical innovations. An appropriate textbook can make clinical physicists interested in the MRI technology up to speed and expedite the translation of the latest MRI methods into clinical practice to improve patient care.

